# Integration Experiences of Former Afghan Refugees in Australia: What Challenges Still Remain after Becoming Citizens?

**DOI:** 10.3390/ijerph181910559

**Published:** 2021-10-08

**Authors:** Omid Rezaei, Hossein Adibi, Vicki Banham

**Affiliations:** The School of Arts and Humanities, Edith Cowan University, Joondalup, WA 6027, Australia; h.adibi@ecu.edu.au (H.A.); v.banham@ecu.edu.au (V.B.)

**Keywords:** Australian society, Afghan refugees, emerging community, integration, cohesion

## Abstract

This paper explores, analyses, and documents the experiences of Afghan-Australians who arrived in Australia as refugees and were granted citizenship after living in Australia for several years. This research adopted a mixed method of qualitative and quantitative approaches and surveyed 102 people, interviewed 13 participants, and conducted two focus-groups within its research design. Analysis of data indicates that former Afghan refugees gradually settled down and integrated within Australian society. They value safety and security, open democracy and orderly society of Australia, as well as accessing to education and healthcare services and opportunity for social mobility. However, since the integration is a long process, they are also facing some challenges in this area. Findings of this study show that Afghan-Australians require more support from Australian governments to overcome some of these challenges particularly securing employment within their area of interests and professional occupations that they have qualifications and experiences from Afghanistan. They are also experiencing broader challenges in the area of socio-cultural issues within Australian society. Since the Afghan community is an emerging community in Western Australia, they require more support from local government to enhance their ethnic cohesion and solidarity.

## 1. Introduction

International migration has been growing exponentially. According to the United Nations Global Migration Database [1], the population of international immigrants around the world increased from 153 million in 1990 to 280 million in 2020, suggesting that people are moving to and residing in countries not of their origin. Considering this trend migration is becoming a global issue of concern across the world. There are a few top countries attracting the majority of migrants for settlements, including the United States of America, Germany, Canada, Great Britain and Australia.

Australia, while being known as a “country of migrants”, was not the destination of non-European immigrants for a long time, due to its White Australian Policy (1901 to 1975). Based on this policy, only people from European countries, mainly from Great Britain, were offered migration to Australia [2]. However, following the change of Australian White Policy and the introduction of the Racial Discrimination Act (1975), the composition of Australian migrant population has changed drastically and immigrants from around the world started to be granted visas to settle in Australia [3]. Today, Australia as an open society with growing economic opportunity is an attractive destination for immigrants from different countries. That is why over 200,000 new migrants select Australia as their permanent destination every year. As a result, 30 percent of the country’s 24 million people had been born abroad [4] and almost half of the population was either born in another country or have at least one parent born overseas [5].

This includes thousands of people with refugee backgrounds living in Australia, including people from Afghanistan. A series of tragic events in Afghanistan were the main cause for Afghan refugee to be settled in Australia. The invasion by the Soviet Union in 1979 with civil wars have placed Afghanistan among the top three refugee-producing countries in the world [6]. Today, there are over 2.6 million Afghan refugees living in Iran and Pakistan, and many more thousands scattered in 70 different countries, including Australia [7]. According to the Australian Bureau of Statistics [8], there were 46,799 Afghanistan-born people living in Australia in 2016, the majority of them arrived in Australia as refugees [9].

The presence of Afghan people in Australia dates back to the 1880s when Afghan people and their camels were brought to Australia to explore the dry central regions and outback of Australia. It is estimated that around 3000 Afghans were involved in camel driving work for over sixty years [10]. They played an important role in Australian transport and made tremendous contributions to the development of Australia. The Existing Ghan Train between Adelaide and Darwin is one of the surviving examples of their contributions. Among recent Afghan arrivals in Australia as refugees, there are many successful stories in terms of adjustments and contributions to Australian society. The following are a few examples of these successful Afghan people in Australia:

Dr. Homa Forotan, as a former Afghan refugee, fled Afghanistan with her parents to live as a refugee in Pakistan in the 1990s, and, after a few years, they decided to apply for Australian refugee permanent visas and eventually arrived in Brisbane, Australia, in 2005. Now, she is a cardiologist in Brisbane and believes that the support she received from Australian people and the government helped her to believe in herself [11]. Another example is Yalda Hakim, who arrived in Australia with her parents as Afghan refugees, where she took advantage of her new society to be a successful internationally-known journalist, working with the SBS and BBC. Hussain Sadiqi is also a Hazara-Australian who was the captain of the Afghanistan national team of martial arts. He became a Taliban target and had to flee Afghanistan; he arrived in Australia by a fishing boat with many other refugees in 1999, when he was 18 years old. He spent 6 months in a detention centre and was then granted a temporary protection visa. Today, he is a well-known martial artist actor in the Australian film industry, and he has won several international festival awards for movie fight scenes.

However, in spite of these successful examples of integration of some Afghan refugees into Australian society, there are still some serious challenges for others, even after receiving citizenship. In this regard, some evidence shows that the white Australian Immigration Policy still operates in some form within Australian society [12,13,14], upon which, words like “race”, “different” and “minority” in public discourse refer to the indigenous Australians, Muslims, Arabs, Asians, and Africans [13]. This means that different intersecting factors like race, religion, gender, and class specify one’s status in the “hierarchy of citizenship” in society [15]. This suggests that certain members of society are considered as first-class citizens, while other citizens are regarded as strangers, due to their different racial characteristics from the majority [15]. So, being second-class citizens will deprive members from an upward social mobility and will create serious challenges for them in terms of their integration process.

These challenges have even led to poorer health condition among Afghan refugees compared with the general Australian population [16,17,18]. These issues are real, and this study documents the views of many people from Afghanistan who have gone through the long process, from being refugees to today being Australian citizens, who have been living in Perth for many years. Therefore, this study aims to understand what integration challenges still exist in Australian society for Afghan-Australians and how Australian governments should be aware of these issues and provide relevant support for the Afghan community to overcome these challenges and become valued additions to Australian society.

## 2. Theoretical Framework

Migration studies have a long history in sociology. It began with the study of immigration and its consequences in the United States by the Chicago School of Sociology in the second half of the 20th Century. Scholars, such as Robert E. Park and Ernest W. Burgess, developed an assimilationist theory and called it the Melting Pot Theory [19]. This was the dominant theory in migration and remained as such until the 1960s. However, sociologists seriously challenged the dominant assimilationist trajectory of the Chicago School of Sociology [20]. Therefore, as immigration has become a critical issue around the world since the 1980s, this field has witnessed huge developments in research, conceptual frameworks, and theoretical perspectives.

**Figure 1:**
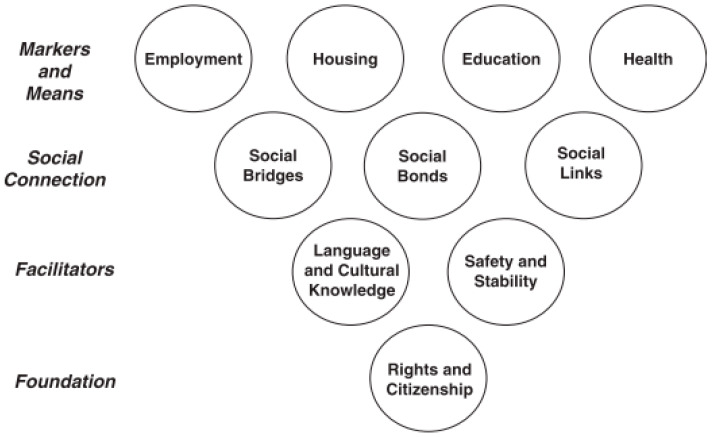
A conceptual framework defining core domains of integration. Reprinted from ref. [21].

This study uses Ager and Strang’s [21] successful integration framework. This framework includes ten core domains of successful integration, which are divided into four areas: markers and means (employment, housing, education, and health), social connections (social bridges, social bonds, and social links), facilitators (language and cultural knowledge, safety and stability), and foundation (rights and citizenship). Figure 1 shows the connections within the core components of integration in Ager and Strang’s conceptual framework (2008).

In this framework, “citizenship and rights”, as a necessary foundation, affects the experiences of immigrants in the receiving society. In addition, the “social connection”, or as Bourdieu (1986) calls it, the “social capital” of immigrants in the receiving society, occupies an important role in the process of integration. Scholars have divided “social connection” into three categories: “social bonds (with family and co-ethnic groups), social bridges (with other communities) and social links (with the host country institutions structures of the state)” [22]. Furthermore, the authors also consider language and cultural knowledge as a necessary element for successful integration. This could not be achieved without the existence of safety and security in a society. This framework is a very useful tool for the use of survey questions, as well as interviews.

There are several recent studies that have applied this model for measuring integration among refugees around the world. Flug and Hussain [23] undertook Ager and Strang’s framework to understand the integration experiences of refugees in the UK, and they found that language barriers, employment challenges, and experiencing racism are refugees’ major problems. In another study that was in the UK and Japan in 2021 [24], Phillimore and colleagues applied this framework to compare refugees’ integration in two research fields. The finding highlighted the importance of social connections in successful integration within both countries. Ziersch and colleagues [25], in another study in Australia, used this model to understand the relationship between integration and social determinants of health. They concluded that challenges of integration can negatively affect refugees’ health and well-being.

## 3. Method

### 3.1. Study Design and Recruit of Participants

In this study, our research design included a mixed methodology to study the Afghan community in Perth. For collecting data, we used a questionnaire, individual interviews, and focus groups. We developed close contact with the Afghan community in Perth by attending various religious and cultural events. Data collection took place in the Perth metropolitan area, Western Australia, between September 2020 and May 2021. The population sample in the survey phase included 102 participants of both sexes, who acquired refugee status first and were then granted Australian citizenships after several years. Within the qualitative phase, 13 participants of both genders were individually interviewed and also 13 individuals participated in two gender-based focus groups. The majority of the interviews and focus group discussions were conducted in the Persian (Dari) language, but English was also used in a few interviews. In studying the Afghan community, the first author of this study, started to contact Afghan community leaders, introducing the research project and asking them to introduce him to research participants. Therefore, the researcher started to attend Afghan religious, social and cultural events all across Perth, to recruit participants for the research survey, as well as to invite them to participate in interviews and focus groups.

Convenience and snowball sampling were conducted in the quantitative and qualitative data collection phases, respectively. Semi-structured individual interviews were conducted within safe and secure venues, including two campuses of Joondalup and Mt Lawley of Edith Cowan University in Perth. Other interviews and focus groups took place at participants’ preferred locations across Perth. Two focus group sessions, with 8 and 5 members from the male and female groups, respectively, were also conducted in Kings Park and Riverton Library in Perth. At the start of the interview and focus group, a consent form was discussed with participants, and they gave their consent, either in writing or orally.

This research was conducted according to the National Statement on Ethical Conduct in Human Research, as well as the Edith Cowan University’s Conduct of Human Research Ethics Policy (approval number 2020-01617).

### 3.2. Study Measures

In conducting the survey, a short one-page questionnaire in both Persian/Dari and English languages was developed and distributed among respondents by the first author of this study, who is fluent in both Persian and English. The first part of the questionnaire assessed demographic characteristics, including age, sex, education level, employment status, length of stay in Australia, ethnicity, and religion. In addition, the second part of the questionnaire was designed based on Ager and Strang’s integration framework, according to which all ten domains of integration were evaluated via ten questions.

Subsequently, semi-structured individual interviews and focus group discussions were conducted based on the conceptual framework. In this regard, the researcher introduced himself and explained the aims of the interview and then tried to ask general questions about different domains of integration to encourage participants to share their experiences of integration into Australian society in the format of Ager and Strang’s integration framework. In the focus groups, a similar structure was repeated and questions raised by the first author and participants contributed through discussion. All interviews and focus groups were transcribed and read several times to reach data immersion. The first author was responsible for coding, which was subsequently reviewed by other authors. After discovering no new data and achieving data saturation, the process of collecting qualitative data was stopped. Then, the transcribed interviews were analysed using thematic analysis.

### 3.3. The Participants’ Profile

There were 102 respondents in the quantitative phase of the study, 54 percent were men and 46 percent were women; almost half of them were born in a country other than Afghanistan, mainly Iran and Pakistan (44% together). Regarding religion and ethnic affiliation, the majority of them are Hazara (65.4%) and Muslim-Shia (80.8%). The average number years of living in Australia for survey respondents, interview participants, and focus group members of this study were 8.5, 7.2, and 9.8 years, respectively. The participants had been living in Australia for an average of 8.5 years. Table 1 shows the demographic information of the participants.

The survey demographic information showed that 21.2% of participants were illiterate in their native Dari language, two-third of them were women. Additionally, only 27.3% of those who had a “4-years degree”, as well as none of those who had master’s degrees, were women. Moreover, the majority of respondents were from the Hazara ethnic group, which is in line with the proportion of total Hazara people in Australia. Data also show that about 51 percent of respondents spoke both English and Persian/Dari during the day, whereas 33.3 percent usually spoke only Persian/Dari. Nevertheless, Persian/Dari was more convenient for 79 percent of them.

Within the qualitative phase, 13 participants were recruited for individual interviews. The lengths of the interviews were between 45 and 90 min long. Eight interviews were conducted with men and five with women, one of which was conducted via telephone. Their age range was between 19 and 62, most of them were in their 30s, the majority were first generation immigrants and identified as Muslim. In addition to individual interviews, there were two focus group sessions, one for men with 8 participants and one for women with 5 participants. The average length of the focus group sessions was approximately 2 h.

## 4. Quantitative Findings

The descriptive statistics of the main variables in the survey showed that the majority of respondents, 86.9 percent, “definitely” or “most probably” wanted to live in Australia for the rest of their lives, and only 1.6 percent wanted to move to Afghanistan if it becomes a peaceful country. This is not surprising, because the situation in Afghanistan has been unstable over the past four decades; particularly, at the time of data collection, the U.S. declared that it was planning to withdraw its troops from Afghanistan. Therefore, returning home to the country was not an option for participants at the time of responding to this survey.

According to the findings, most participants, 56.4 percent, considered both Australia and Afghanistan as their homelands, and 20 percent considered only Afghanistan and 21.8 percent chose Australia as their homeland. Our findings also showed that the respondents were not interested in following Afghanistan’s news and only 40.4 percent of Afghans mentioned that they “sometimes” follow the news from Afghanistan media. As many Afghan refugees still suffer from the war-trauma-related issues, it can be a form of “self-care” for participants to hear less bad news from their home country.

Table 2 demonstrates the main findings regarding the different domains of integration among the research respondents. Based on the theoretical framework, all the research participants possessed the foundation of integration, because they were all Australian citizens. After several years of living in Australia, they also did not seem to have major issues in some areas, such as housing, education, health, and safety. However, the findings indicated that they had challenges in some integration domains, such as employment and social connections, as well as language knowledge and proficiency.

According to the findings, 54.8% of respondents were working, two-third were men. In addition, the majority of unemployed respondents, 83.6%, as well as all those who worked as unpaid volunteers and houseworkers were women. Findings on employment also indicated that the level of education among more than 80% percent of unemployed Afghans was “high school” or lower. In addition to this, the mean of respondents’ satisfaction with their current employment was 42.5 out of 100, which is lower than the average.

In addition to employment, social connections are another domain of integration in which respondents had the lowest scores compared to other domains. Afghans’ social connections are divided into two categories: connections within their community and connections outside their community. Although their connections outside their community were weak, particularly among those who declared having English language barriers, surprisingly, even their social connections within the Afghan community were also weak. Finding shows that the weakest connections with Afghan community were among those who declared that they have “no religion”, as well as those who were born and lived in Iran. The majority of Afghan community members believe in Islam, more than 90 percent of the respondents, and their connections usually occur during Afghan religious events and gatherings, where non-religious Afghan members do not participate and consequently miss the opportunity to strengthen their social connections. Ethnic identity is also another important factor in creating inner-group social connections within the Afghan community, but these ethnic divisions do not exist for the Hazara people who came from Iran.

In the area of language proficiency, the majority of respondents who had the lowest level of English proficiency were Afghan women, as well as those with lower education levels. Based on traditional Afghan gender roles, women are usually responsible for managing domestic chores, while men work outside. Due to this fact, women’s opportunities for improving their English language are limited. This is also true of participants with lower education levels who cannot improve their English proficiency by developing their education.

## 5. Qualitative Findings

**Figure 2:**
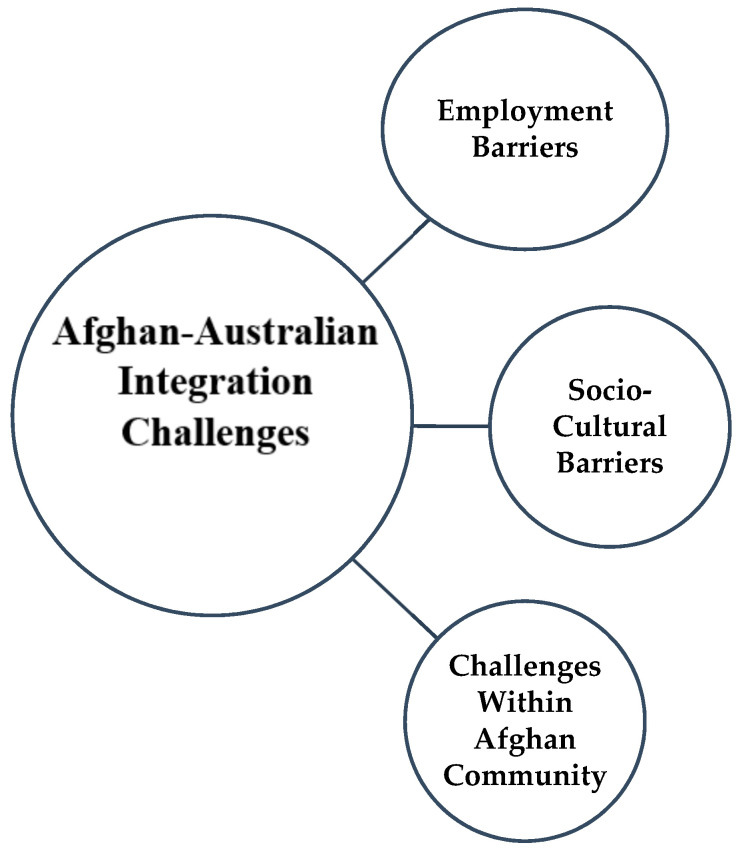
Main themes that emerged from qualitative data.

Undertaking a bottom-up approach in thematic analysis, the researchers started with transcriptions, going through the data and noting similarities in the interviews. In this regard, dozens of simple codes were created and, in following step, they were grouped together to find patterns in the data. To do this, researchers drew a map of codes to understand the relationship between them in order to create themes. Finally, three main themes emerged from the qualitative data, indicating the main challenges for former Afghan refugees in Australia: employment barriers, socio-cultural barriers, and challenges within the Afghan community in Perth. These challenges can be categorised under integration markers and means, as well as social connection (within and outside the Afghan community). Figure 2 shows the themes.

### 5.1. Employment Barriers

For many participants, finding a job that is related to their skills and qualifications was very difficult. A part of this difficulty relates to problems with recognition of overseas qualifications in Australia. Sadiq is a participant who came to Australia as a refugee in 2015 and was awarded an Australian citizenship recently. He holds a bachelor’s degree in chemistry from Kabul University and worked as a high school teacher in Afghanistan, but Australia does not recognize his qualification. In this case, he has to enroll in university courses again, or change his field of study.

*“They don’t recognise my qualifications here. So, I have to study 8 years if I want to be a teacher [in Australia], but I don’t have time. I have to work and make money to support my family in Afghanistan. That’s why I’m working in construction field now.”*

In this regard, several studies have shown that a significant relationship exist between employment-related difficulties and recognition of qualifications in Australia [26,27]. As a result of this barrier, the process of employment is a highly challenging factor for many Afghan immigrants, and not all of them are able to either negotiate their situation or afford to continue tertiary studies in Australia.

Reza is a refugee; when he arrived in Australia as a refugee in 2015, he had a Bachelor’s degree in sociology from Afghanistan, along with a few years of work experience with an international organization in Afghanistan. In spite of his university degree and work experience, as well as his good command in English proficiency, he was not able to find a job during his first year living in Australia. He discussed two main reasons for his failure and said: “they didn’t recognise my qualifications and also all jobs require local experience”. Even after graduating from an Australian university, he ended up working in a field that was not his first choice.

*…so, I had to go to university again but how? I needed the IELTS certificate, as well as paying the tuition fees… Finally, I graduated from a university, but I still wasn’t able to find a job in my favourite field [International Security Studies], so, I had to shift to the field of Social Work and finally could find a job after a couple of years.*

Discrimination in the process of the finding a job, as well as in the workplace was another challenge that many Afghan participants face in Australia, regardless of their citizenship status. According to Dion [28], immigrants may experience discrimination because of the place they came from or they may be seen by others through their skin colour, language, or religion. Zari, as a Hazara woman, is an example. She is in her 20s and mentions that she has been facing many barriers in finding a job, because of her hijab.

*“I haven’t been able to find a job mainly because of my Hijab. Even some employers have said this to me directly. My uncle is an owner of a business in Perth, but even he doesn’t hire me for my Hijab... That’s why I have to look for a job only in Afghan community.”*

However, discrimination in the workplace is not merely limited to the hijab. Sanam, another female participant, who does not wear Hijab but still faces the same barriers. She introduces herself as a modern lady who respects the modern values of Australian society and has limited connections with the Afghan community, mainly “because of their traditional mindset”. Nevertheless, she has several stories about discrimination in career promotions. Here is one of them:

*“I had an Australian employer that was telling my colleague that if she [the participant] was paler, I would use her in the other section [which was more important]. I heard it myself... You are a foreigner in the workplace, no matter how much you try to be similar to them.”*

Nevertheless, some participants view this discrimination in a wider perspective. A participant who had been living in Australia for almost 30 years believes that discrimination in the workplace is not limited to only to people with an Afghan background, but it is extended to all non-white people in Australia in general. Naser shared:

*“When you look at the Australian government, from parliament to governmental organizations, the absolute majority of employees are those with White background. So, non-white people can only pursue their employment in non-governmental sections. That’s why you see lots of businesses in shopping centres with Asian or African owners. In fact, the multicultural Australia is only represented in shopping centres not in government or public organizations.”*

Participants also discussed more issues and challenges within the employment area, such as language barriers, lack of job security, and exploitation in the labour market. While participants discussed challenges, they also discussed some of their plans and strategies that they will employ to secure their employment in the future. Some of the changes include changing their names to Anglo-Australian names. They were also very serious to further enhance their English proficiency as a key factor in securing relevant employment and building a proper career.

### 5.2. Socio-Cultural Barriers

Former Afghan refugees have been facing several challenges in the context of socio-economic aspects of their lives in Australia, one of which is the problem of revealing their identity as Muslim-Afghans in Australia. Due to negative feedback that most of participants received from Australian society about Afghanistan, mainly after 11 September 2001, they were uncomfortable with expressing their Afghan identity while communicating with people outside of the Afghan community. Ali and Saeed told their stories about hiding their Afghan background since they had received discriminatory reactions. They share:

*“At first, sometimes people would ask me: where are you from? And I would say: I am from Afghanistan. Then they would say: Oh, Taliban. Or they’d say do you know Osama Bin laden? So, I realised that I don’t have to tell them the truth. Since then, whenever somebody asks me where are you from? I say Tajikistan or Uzbekistan.”*

(Ali, 39 years old)

*“My younger brother is a student at […] University. One day I was working with him in a construction project and the man that we were working for asked my brother what do you study? My brother replied: piloting. Then the man said: “oh. Okay, you plan for hijacking”. It really made me sad and for a couple of days and decided not to tell people my nationality anymore.”*

(Saeed, 41 years old)

As a result, after the tragic events of 11 September, Muslim immigrants have been targets of discrimination in Western countries and this has led them to hide their identities. This particularly makes them feel that they are seen as “others” in these societies.

In addition to Muslim-Afghan identity, some participants were not comfortable with their former refugee identity. This is mainly because Afghan refugees in Australia are known as “boat people”, which refers to the way in which they came to Australia. However, there is a generational difference between participants in accepting their refugee background. Younger participants tend not to talk much about their refugee status in the past, while older participants easily talk about being refugees sometimes in the past. These older participants also complained about the way that Australian people and media view refugees. During las decade, Australian media negatively focused on Afghan refugees who arrived in Australia by boats and called them “boat people”. Sara is in her 60s and has been living in Australia for over 30 years. She had a pharmacy in Afghanistan in the 1970s, but had to flee Afghanistan as a result of the Soviet Union invasion in the 1980s. She shared:

*“It’s always been so difficult for me to explain people that we were fleeing from violence and war. We didn’t want to leave our country voluntary, but we had to do that. People here don’t know anything about war, violence… they just blame refugees for coming to Australia…if they know a little bit more about refugees, their attitude will be changed.”*

Another social barrier that was mentioned by many participants is that they are not able to broaden their social network to include other Australian people. Several studies have also shown that there are challenges for young immigrants in developing their social network within the Australian multicultural community [29,30]. This is even true about participants that have been trying to be assimilate into their new society. Sanam and Zari shared:

*“No one can understand from my appearance that I am Afghan or Asian. No matter how you dress like them or how good your language is, people believe that you have come from a foreign country…Finding an Australian friend has been so difficult for me.”*

(Sanam, 24 years old)

*“I can’t find any non-Afghan friend here, whenever I try to communicate with a guy, after a while, either I find them different or they do. That’s why I’ve stopped looking for a friend outside the Afghan community.”*

(Zari, 28 years old)

Some participants also revealed some challenges that they faced with their children who are the second-generation Afghans. First generation Afghan refugees would like to maintain their culture, religion, and language, while their children are reluctant to accept these values. The gap between the two generations has become bigger, in the case of parents who have not spent enough time helping their children to learn about their culture and language.

*“My son is completely different from me. I speak Dari with him but he replies in English. But for me, the most important thing is religion. I am not so strict about language…I tell him if you speak two languages, that would be an advantage, but if you don’t believe in Islam, that’s a shame.”*

(Naser, 51 years old)

*“I have several Afghan friends who are really hardworking…they haven’t taken the time to help their children learn to speak Farsi. Now, their children speak English with them and they can’t understand them. I think it is a really sad story, because your kid is the most valuable thing that you have in the world.”*

(Saeed, 41 years old)

### 5.3. Challenges within Afghan Community

In addition to facing the above barriers, former Afghan refugees face some challenges within their own community in Perth. One of these challenges to is related to the fact that Afghanistan is a multiethnic society and peoples have been involved in ethnic divisions for a long time. Unsurprisingly, these divisions have affected the Afghan diaspora in Perth as well. Thus, there is no single Afghan community in Perth, rather there are several. Each ethnic group has its own members in terms of organising social or cultural events. Mehdi, who has been living in Perth since 1992, has witnessed the changes that happened in the Afghan community over time.

*“In those first years the number of Afghans in Perth was low; ethnicity was not so important and there was a single community. But after arriving more Afghan refugees in the following years, and especially after establishment of Taliban and deteriorating the ethnic divisions in Afghanistan, the situation in Afghan community in Perth changed. So, every ethnic group started to build a community for itself. Today, there are several of them which are independently active.”*

These divisions are even strengthened by religious differences that exist within Afghan ethic groups. Afghan Hazaras are mostly Muslim-Shia, while other groups like Pashtuns and Tajiks are Muslim-Sunni. Thus, this has created some challenges for those former Afghan refugees who care about their religious belief. Naser shared:

*“We [Shia] used to have a problem when someone from our community died, because we didn’t have a place to wash a corpse before burying and we had to ask [Sunnis] to use their place. But they sometimes refused, because they don’t respect the way that we wash a corpse, and would say it is not recognised in Islam.”*

Another challenge that several participants mentioned is that most Afghan community events and gatherings are religion-based, which is due to the mindset of Afghan community leaders. This factor may prevent people with different belief systems from being attracted to that community. Saeed is one of these people, who shared:

*“Almost all events are about religious things. I tried to add some other programs, like Persian Poetry reading program for some passionate people that I knew, but those narrow-minded people didn’t allow us to continue our program. They would suggest run a Quran class instead…. I even tried to run some events independently, for example I managed to celebrate the Women’s day last year, but those leaders have a huge influence among Afghans, and they’d advised people not to join the program and finally we run the program with only a few women.”*

Many Afghan refugees, before arriving in Australia, lived in neighbouring countries of Pakistan and Iran, for many years. Some of them even were born in these countries and have never been in Afghanistan. This has added more complexity to the Afghan community in Perth. Thus, in addition to ethnic/religious divisions, there is another classification division based on the country that Afghan refugees have lived in before coming to Australia. Hamed is a 34-year-old Afghan-Australian who was born in Iran. Checking his Facebook profile before conducting an interview, the researcher realised that he is a big fan of football, and surprisingly there was the flag of Iran in his cover photo. He has never been in Afghanistan and is not interested in Afghanistan issues. Instead, he tries to talk more about Iran than Afghanistan during his interview. He shared:

*“I am not much in contact with Afghan community here, we are not similar, to be honest. I rather have a few Iranian friends and that’s enough… Those Afghans who’ve come from Pakistan also have their own connections with Pakistani people, and it is true about those who have come from Afghanistan directly.”*

Moreover, there have been some changes within Afghan families after migrating to Australia that created some new challenges, specifically for Afghan women. These challenges mainly are caused by the culture of masculinity, as well as by the changing of the gender roles in the family. In Afghanistan, a man/husband is recognised as a bread winner and decision maker within the family and all family members usually follow his orders. In addition, there is a traditional definition of gender roles in which a man is responsible for work outside, while woman is expected to manage domestic chores. However, after migrating to a country like Australia, which does not recognise these definitions, the dominant position of father, as well as other male gender roles within the Afghan family has been gradually changing. In this regard, there are still issues in relation to power distributions within the family. For example, husband is expected to make decisions on almost every aspect of family affairs and activities, including controlling their income and their relationships outside of family and even father can make decision on to whom his daughter should marry. Since these are in contrast with the values of a modern society like Australia, it may lead to tension within the family. Zari is an Afghan-Australian who has been living in Australia for almost 8 years. She shared:

*“A few years ago, I started to work at […], but all my income was controlled by my husband. I even didn’t know that how much is my income and how is being spent in our life. But after a while I started to complaining and he was responding by limiting my freedom. Since then, I couldn’t go to my friends’ gatherings.”*

In addition to financial issues, there are other challenges that Afghan women are facing and dealing with. Female participants shared stories about their experiences and other Afghan women that they know who have had similar experiences. Zari shared:

*“A friend of mine who is a young lady, has a strict Afghan father who imposes lots of restrictions on her relationship with others, particularly with boys. So, what she does is that she goes out with her boyfriend from 11 pm to 6 am, when her father is asleep….my other friend has been forced to marry with a relative who lives in Afghanistan; they’ve never met each other.”*

## 6. Discussion

Adjusting to a new society is not an easy journey for everyone, particularly for refugees who may have not been ready for this change. However, the host society can assist refugees to have a successful integration into their new society. The participants of this research, who are former refugees, have already been provided with support by the Australian government, which means that they have the same rights as other Australians. That is why the majority of the participants consider Australia as their homeland and about 90 percent of them would like to live in Australia for their rest of life.

However, this study showed that while former Afghan refugees enjoy some aspects of Australian society, at the same time they face some serious challenges in their new society. Challenges include employment, socio-cultural atmosphere of society, as well as socio-religious barriers within the Afghan community in Perth, Australia. This is evidence showing that, while they are legally Australian citizens, they are not fully accepted as members of society. This is in line with Lazarus’s research that “some citizens more equal than others” [15].

Nevertheless, according to the quantitative findings, only 1.6 percent of Afghan participants want to return to Afghanistan, which means that, in spite of these challenges, they still prefer to live in Australia. Now that Afghanistan has been taken over by Taliban as of 15 August, 2021, the situation of Afghan refugees around the world has changed drastically; according to the UNHCR, over 550,000 Afghans have been forced to flee their homes and seek refuge in other countries. Therefore, returning to Afghanistan is not an option for Afghans living in Australia at the present time.

The experience of four decades of war in the participants’ home country is still alive in Afghans’ memories. All these have generated poor health conditions, as well as psychological issues for them, including concerns about safety and post-traumatic stress disorder [31,32]. This was even obvious in interviews with participants, where most of them reported losing someone from their family, or friends in the war in Afghanistan. That is why, according to our quantitative findings, safety and stability were perceived to be the highest score among the domains of integration. This is in line with research that was conducted by Abedin [33] in which safety was one of the main reasons affecting Afghan women’s sense of belonging in Swedish society. Nevertheless, considering the traumas they have experienced, as well as possible health problems, many Afghan-Australians need to be supported in terms of mental health and psychological services, especially by services that are culturally sensitive.

However, at the top of the list in our research, employment stands as the biggest challenge for Afghan-Australians. As one study notes, “employment is the most common means through which people can express their creativity and find ways to fully participate in society” [34] (p. 61). However, as some studies have shown, there exists a large inequality between the earning and income of migrants as compared to the rest of the population in Australia [35,36]. According to the 2016 Census, the average weekly income for Afghanistan-born people is $371, while the median weekly income for Australian-born residents is $688, which represents a significant difference [8]. The census also reported a rate of unemployment among those who were born in Afghanistan of 17.8 percent, which is far from the 6.9 percent unemployment rate for all Australians. Several studies have discussed the challenges that immigrants have been facing in Australia, indicating the prevalence of overt and covert discrimination in the Australian labor market (e.g., [18,19,20,21,31]). It seems that COVID-19 has worsened the situation for migrants in Australia, which is beyond the scope of this paper.

In addition, socio-cultural challenges reported by participants can impact their integration within Australian society. A big part of these challenges is related to the discrimination that Afghan-Australians perceive from society, in which they are often identified like “terrorist militants”, and not as victims of terrorism. Nevertheless, as is discussed in this paper, these barriers can be, on the one hand, structural barriers like racism, discrimination, and lack of social capital in the host society or, on the other hand, ethno-religious factors, such as parental disapproval of participation in particular events or because of different definitions of gender and social roles [22]. As a result, while there are structural barriers within Australian society that narrows migrant social networks, in many cases an immigrant’s ethno-religious beliefs also do not allow them to easily communicate with other people outside their cultural boundaries.

An interesting finding in this paper is related to the challenges that former Afghan refugees face within their Afghan community in Perth, Australia. According to Daley [37] refugees’ community support can play a vital role in the development of social bonds, bridges, and links that are key indicators of integration in the host society. However, ethnic and religious tensions in the Afghan community deprived members of having a unified community in Perth. This is not limited to the Afghan community in Perth, however, as Abraham and Busbridge [38], in their research, reported the existence of similar tensions among the Afghan community in Melbourne as well. This lack of cohesion in the Afghan community not only limits social interactions for community members with the wider community in Australia, but also prevents them from having a unified voice to represent community needs to the Australian government in order to receive more support.

## 7. Conclusions

During the recent migration of Afghan people to Australia, despite the fact that migration has been a complex and difficult change for them, in this journey, Afghan people have been successful in establishing thriving communities across Australia. This research discussed distinctive characteristics of the Afghan community in Perth, including their contributions and connections to Australia, as well major challenges. Afghan refugees have found Australia to be their new home and the overwhelming majority of them consider Australia as their permanent country of residence and only 1.6 percent of the participants indicated that they may return to Afghanistan if it is safe in their replies.

However, in spite of the Australian government’s support in granting many Afghan refugees citizenship, they still cannot enjoy the full advantages of citizenship rights in practice. The Afghan community in Perth, with the strong commitment to make Australia as their permanent home, strongly needs and deserves to receive the Australian government’s support. In addition, the inclusion of Afghan people within the broader Australian society facilitates the integration process and the media can play a vital role in developing the local community’s awareness about refugees, their backgrounds, as well as their potential contributions to Australian society. This social inclusion is particularly crucial for further enhancing the quality of the Australian multicultural society. To do this, conducting more research on some aspects of Afghans’ lives in Australia, particularly in the context of employment, as well as their relationship within the Afghan community, would be helpful.

However, in this difficult time, where the Taliban have returned to power in Afghanistan, and crisis after crisis has been happening in Afghanistan, the Afghan community in Australia also has been experiencing a very difficult time along with high anxiety and so many facing unwelcome news; it is time that the Australian government provide urgent counselling services with cultural sensitivity to those who desperately need them. These services should be part of an “ethnic particular service”, and not a mainstream service. Afghans are highly concerned about the future of their country, the lives of their family and relatives in Afghanistan, as well as uncertainty of many Afghan refugees with temporary protection visas in Australia. While at the present time, the Australian government has allocated 3000 out of its 13,750 annual humanitarian visas to those fleeing Afghanistan, the government can do more. Therefore, in line with over 300 Australian organizations, businesses, and community groups that signed an online letter, demanding from Australian government to assist the Afghan community, recommendations are listed as follow:

1. Assist Afghan people who are at grave risk in Afghanistan, particularly those who have worked with the Australian government by facilitating evacuation.
2. Dedicating more humanitarian visas to Afghan refugees to protect lives at risk. These can be announced as special refugee visas.
3. As millions of people in Afghanistan have recently become displaced, many are at risk of hunger and lack of shelter. Thus, the Australian government can increase its aid to the region, for example, by supporting organisations that assist people in Afghanistan.
4. Extend permanent protection to over 4000 Afghan refugees on temporary protection visas in Australia, as it is not safe for them to return to Afghanistan.
5. Assist Afghan-Australians with urgent family reunion visas, particularly for those who are at greater risk, including ethnic minorities and relatives of those who have worked with the Australian government in Afghanistan.
6. Taking more Afghan refugees, the Australian government can take advantage of this opportunity to provide workforces to Australian regional areas that are in desperate need of workers, mainly as a result of closed borders from the COVID-19 pandemic.

## Figures and Tables

**Table 1:** Participant demographic information.

Demographic Information	Categories	Survey (N = 102)	Interviews (N = 13)	Focus Groups (N = 2)
Gender	Men	54.2%	8	8
	Women	45.8%	5	5
Education Level	Illiterate	21.2%	2	3
	High school	30.8%	4	5
	Some college	9.6%	0	0
	2-year degree	13.5%	3	2
	4-years degree	21.2%	2	3
	Master’s degree	3.7%	1	0
	PhD	0	1	0
Ethnicities	Pashtun	1.9%	2	1
	Hazara	65.4%	7	9
	Tajik	11.5%	1	1
	Sadat	8.5%	2	3
	Others	12.7%	0	0
Religion	Islam-Sunni	11.5%	3	1
	Islam-Shia	80.8%	8	12
	No religion	7.7%	2	0
Average years of living in Australia		8.5	7.2	9.8

**Table 2:** Integration domain key findings.

Integration Domains	Key Findings
Employment	Status: 54.8 on ‘paid work’, 19.1 % ‘student’, 12.2% ‘unemployed’, 5.2% ‘unpaid voluntary work’, 8.7% ‘unpaid housework’ Mean of satisfaction with current employment: 42.5 (out of 100) Mean of qualification’s matching with current job: 58.5 (out of 100)
Education	Mean of satisfaction with Australian education system: 81.8 (out of 100)
Health	Mean of satisfaction with Australian health system: 59 (out of 100)
Housing	Mean of Satisfaction with housing in Australia: 62.6 (out of 100)
Social Connections	Mean of social connections within Afghan community: 32.2 (out of 100) Mean of social connections outside the Afghan community: 30.1 (out of 100)
Language knowledge	Self-reported level of English proficiency: 38.2 (out of 100)
Safety and Stability	Self-reported sense of safety and stability: 92.4 (out of 100)

## Data Availability

Not applicable.
